# Alternative Spliced CD1D Transcripts in Human Bronchial Epithelial Cells

**DOI:** 10.1371/journal.pone.0022726

**Published:** 2011-08-11

**Authors:** Kambez Hajipouran Benam, Wai Ling Kok, Andrew J. McMichael, Ling-Pei Ho

**Affiliations:** MRC Human Immunology Unit, Weatherall Institute of Molecular Medicine, University of Oxford, Oxford, United Kingdom; Karolinska Institutet, Sweden

## Abstract

CD1d is a MHC I like molecule which presents glycolipid to natural killer T (NKT) cells, a group of cells with diverse but critical immune regulatory functions in the immune system. These cells are required for optimal defence against bacterial, viral, protozoan, and fungal infections, and control of immune-pathology and autoimmune diseases. CD1d is expressed on antigen presenting cells but also found on some non-haematopoietic cells. However, it has not been observed on bronchial epithelium, a site of active host defence in the lungs. Here, we identify for the first time, CD1D mRNA variants and CD1d protein expression on human bronchial epithelial cells, describe six alternatively spliced transcripts of this gene in these cells; and show that these variants are specific to epithelial cells. These findings provide the basis for investigations into a role for CD1d in lung mucosal immunity.

## Introduction

CD1d is a member of the CD1 family of transmembrane glycoproteins. It presents self and foreign glycolipid antigens to a group of T lymphocyte population called natural killer T cells (NKT cells) [1]. In this context, CD1d shows high structural homology to the MHC class I genes which encodes a type I integral membrane protein (α heavy chain) with three extracellular domains: α1, α 2, and α3 [1]. Like HLA A–C, CD1d protein non-covalently associates with β_2_-microglobulin (β_2_m); but unlike the MHC gene, its expression on professional antigen presenting cells (APC) is limited in genetic and allelic variation, and is thought to predominantly engage the semi-invariant T cell receptor (TCR) expressed by NKT cells. However, the consequence of this interaction is distinct and wide-ranging. When activated, NKT cells produce large amounts of cytokines (including TH1, TH2 and TH17- related cytokines) [2], [3], [4], [5], and much more rapidly than do conventional T cells. They have the capacity to critically modulate immunity by interacting with T cells, NK cells, macrophages and B cells. They regulate the development of a number of inflammatory diseases – best shown in animal models of Type I diabetes mellitus, multiple sclerosis, asthma and infectious diseases [6], [7], [8], [9], [10], [11], [12].

In the lungs, divergent effects have been noted after NKT cell activation. We have shown that NKT cells participate in immune responses in the lungs, and are protective during influenza virus infection [11], [12]. Their numbers increased in the lungs within three days of influenza virus infection, and activated NKT cells can amplify the innate immune response and decrease early viral load. De Santo *et al* also showed that NKT cells can reduce the recruitment of ‘myeloid-derived suppressor cells’ which allowed increased proliferation of influenza virus-specific CD8 T-cell responses [11]. CD1d-deficient mice that lack NKT cells also succumb to other bacterial infection of the lungs like pseudomonas aeruginosa [10]. However, NKT cells could also be pathogenic in non-infectious conditions – for example in the development of airway hyper-responsiveness in animal models of asthma [9].

Despite these studies, it is not clear how NKT cells arrive in the lungs – whether, they migrated there, if they proliferated in the lungs, and how they are activated in the lungs. Intra-nasal administration of a glycolipid ligand showed a quick expansion of the cells in the lungs, suggesting that a CD1d-expressing cell population could present antigen to NKT cells in this site [12]. One possibility is dendritic cells, which are known to express CD1d, and found in small numbers within the airways. Another possibility is the airway epithelium. Potentially, CD1d expression on bronchial epithelium could be involved in activation of NKT cells or promote engagement of NKT cells with bronchial epithelium and enhance the role of bronchial epithelium in mucosal immunity. To date, CD1d expression has not been observed in primary or bronchial epithelial cell lines but it has been reported on epithelial and parenchymal cells in liver, kidney, intestine and skin [13], [14], [15], [16], [17]. The function of CD1d on these structural cells is not clear, although freshly isolated intestinal epithelial cells pulsed overnight with a glycolipid were capable of activating NKT cells [18]. Here, we provide the first report on CD1D mRNA and CD1d protein expression in human bronchial epithelial cells, and describe six alternatively-spliced transcripts of this gene in these cells. This provides a basis for investigations into a role for CD1d in lung mucosal immunity.

## Results

### Primary human bronchial epithelial cells and airway epithelial cell lines express CD1D

**Figure 1:**
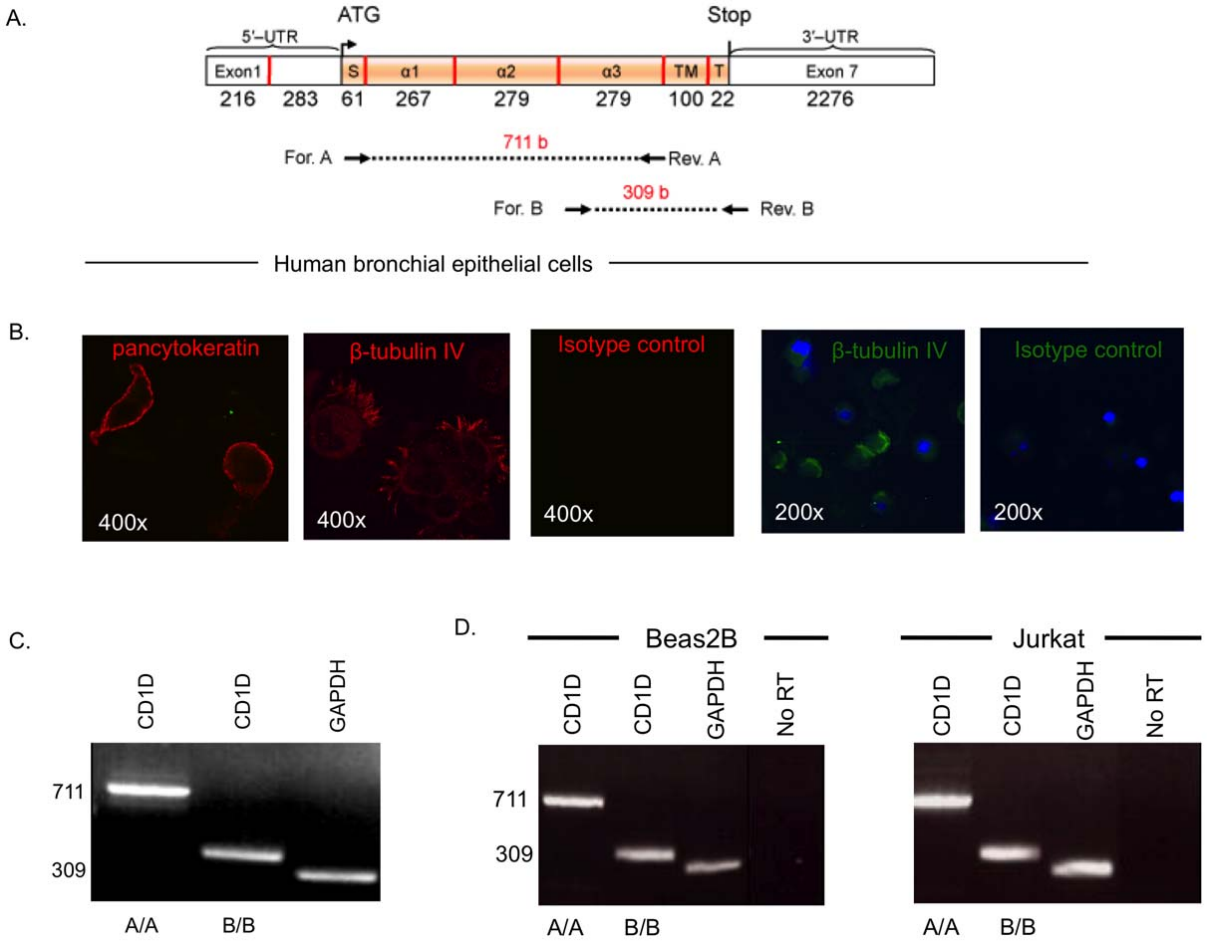
Identification of CD1D transcript in human respiratory epithelial cells. Panel A: Graphical representation of CD1D mRNA; coding exons in orange and non-coding exons in open boxes. Primer annealing sites are illustrated by horizontal arrows, with the expected amplicon size for each pair. Panel B: Brushed bronchial cells immunostained with pancytokeratin and β–tubulin IV, identifying epithelial cells and cilia respectively; “isotype control” - no primary Ab treatment. The 200× magnification shows ß tubulin IV staining in green and nuclei in blue (DAPI). Panel C: Agarose gel on the right demonstrates RT-PCR on these cells. Panel D: CD1D expression on Beas2B and Jurkat cell lines.

Three sets of primers (“A/A”, “B/B” and “C/C”) were designed to specifically amplify CD1D transcript within its coding exons (Table 1, and Figure 1A). “A/A” spanned α2 exon covering most of α1 and α3 exons, and “B/B” amplified transmembrane (TM) and most of α3 and cytoplasmic tail (T) exons. We first determined if human bronchial epithelial cells expressed CD1D, using RNA derived from ex vivo primary human bronchial epithelial cells obtained by bronchoscopic brushing. These brushed cells comprised 90% epithelial cells, as shown by pancytokeratin and ß tubulin IV immunofluorescense staining (Figure 1B), and were found to express CD1D (Figure 1C, right panel). Since CD1D-expressing cells may have contaminated this population of primary epithelial cells, we proceeded to examine CD1D expression in pure airway epithelial cells using the Beas2B cell line. RT-PCR detected CD1D transcripts in Bea2B (Figure 1D). The Jurkat cell line, which naturally expresses CD1d protein, was used as a positive control. Absence of contaminating DNA was shown by including a reaction where no reverse-transcriptase was used (‘No RT’).

**Table 1:** Primers and probes used in this study.

Target gene	Primer or Probe sequence (5′→3′)
**RT-PCR**	
*CD1D*	For. A: CTCGTCCTTCGCCAATAGC
	Rev. A: ACCACATCCAGGGTTGCTC
	For. B: AGCCTGTATGGGTGAAGTGG
	Rev. B: GGACGCCCTGATAGGAAGTT
	For. C: CTGCTGTTTCTGCTGCTCTG
	Rev. C: GACTCAAGGAGGCCACTGAC
	For. D: CTGGGAACGCCTCAAATAAC
	Rev. D: ACAGGACGCCCTGATAGGA
	Rev. E: GAAAGCTGCCTCATGACTGTT
*GAPDH*	For. GAGTCAACGGATTTGGTCGT
	Rev. TTGATTTTGGAGGGATCTCG
**qPCR**	
*CD1D*	For. V1 (SYBR): GCGCTGAAGATCCCTTGGA
	Rev. V1 (SYBR): TACATGGAAGAAGTTATTTGAGGCG
	For. V3 (SYBR): CAAGAGGCCCCACTTTGGT
	Rev. V3 (SYBR): GAGACATGGCACACCAGCAG
	For. V4 (Taqman): AACAGTGCAGTGGCTCCTTAATG
	Rev. V4 (Taqman): TCCCACCTTGCTTCTTCAGTTC
	Probe V4 (Taqman): CCCAATTTGTCAGTGGCCTCCTTGAGT
	For. V5 (SYBR): GAGGCCCCACTTTGGGTAAA
	Rev. V5 (SYBR): CAGGACGCCCTGATAGGAACT
	For. V6 (Taqman): TTTGGAGCAGGTGGGAGCTA
	Rev. V6 (Taqman): CGGGAGGTAAAGCCCACAAT
	Probe V6 (Taqman): AGTCCTGGCGTGCTTGCTGTTCCTC
*HPRT*	For. (Taqman): GACTTTGCTTTCCTTGGTCAGG
	Rev. (Taqman): AGTCTGGCTTATATCCAACACTTCG
	Probe (Taman): TTTCACCAGCAAGCTTGCGACCTTGAT

For.: Forward primer; Rev.: Reverse primer; SYBR: real time PCR amplification by SYBR Green method; Taqman: real time PCR amplification by Taqman assay using FAM-, TAMRA-conjugated primers; V1–6: CD1D spliced variants.

### Expression of CD1d protein on epithelial cells was observed with some but not all CD1d mAbs

**Figure 2:**
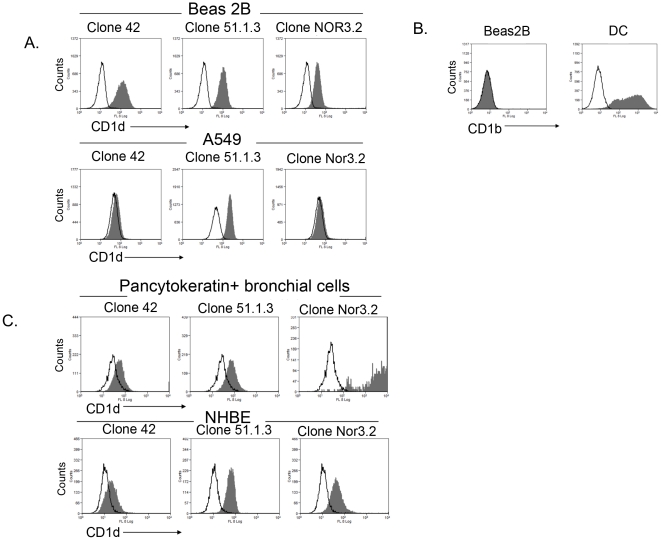
Human respiratory epithelial cells express CD1d protein. Panel A: Flow cytometry analysis of CD1d expression on Beas2B and A549 epithelial cell lines, using three anti-human CD1d mAbs – clones 42, 51.1.3, and NOR3.2. Panel B: CD1b staining on Beas2B cells and CD1b-expressing monocyte-derived dendritic cells (DC). Panel C: Flow cytometry analysis of CD1d expression on primary human lung epithelial cells (top panel) and normal human bronchial epithelial (NHBE; bottom panel) cells.

Using three mouse anti- human CD1d mAbs (clones CD1d 43, CD1d 51.1.3 and NOR 3.2), we showed surface CD1d expression on Beas2B, primary brushed bronchial and NHBE cells with all three mAbs; but, A549 – a Type II human pneumocyte, stained positive with one Ab only (Figure 2A–C). The clone 51.1.3 had been previously reported to partially cross-react with CD1b [19]. To verify CD1d detection, Beas2B cells were stained with an anti-CD1b mAb. We found no CD1b expression on Beas2B, whereas the positive control, monocyte-derived dendritic cells (MD-DCs) distinctly expressed CD1b (Figure 2B).

The epitopes for these CD1d mAbs are currently unknown. However, the differential expression raised possibility of the existence of variants and prompted design of further primer sets to examine this prospect.

### RT-PCR of primary bronchial epithelial and cell line confirms expression of CD1D in bronchial epithelium and reveals six variant transcripts

**Figure 3:**
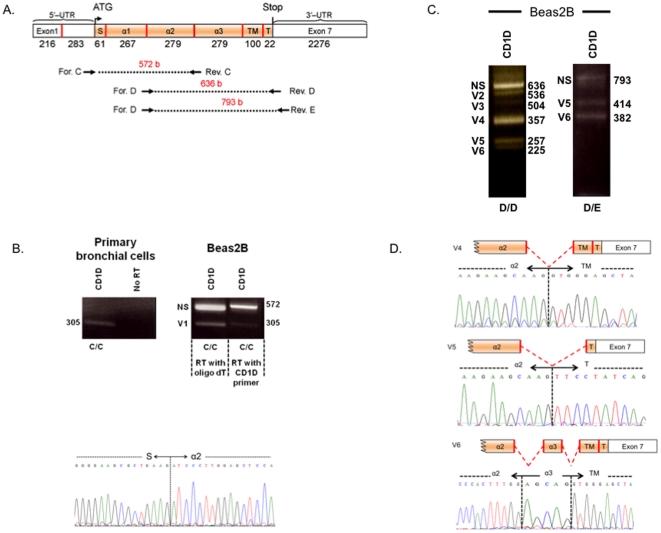
Expression of six CD1D alternatively spliced CD1D variants in human respiratory epithelial cells. Panel A: Schematic illustration of primer pairs “C/C”, “D/D”, and “D/E” annealing sites on CD1D mRNA, and corresponding full-length product sizes. Panel B: RT-PCR on primary bronchial epithelial cells detected a 305-bp band. Same primers amplified the expected full-length (572-bp) product plus a shorter amplicon (305-bp) both in oligo dT– and CD1D specific primer–reverse transcribed cDNA from Beas2B. Direct nucleotide sequencing of the smaller band (lower panel) identified a CD1D variant lacking α1 exon (“V1”). Panel C: Primer pairs “D/D” and “D/E” detected five other CD1D variants (“V2–6”) in Beas2B. Panel D: Direct nucleotide sequencing of “V4–6” revealed the splicing junction in these variants.

To explore the possibility of splice variants, primers were designed to probe the exon boundaries (Figure 3A) (primer pairs “C/C”, “D/D” and “D/E”). Using primer pair “C/C”, which amplifies a 572–bp product containing parts of the signal peptide (S) and α2 exons, and the whole α1 exon of CD1D, we unexpectedly, observed a 305-bp product in brushed primary bronchial epithelial cells (Figure 3B, left panel), which raised the possibility of an alternatively spliced variant. This was also the case in the Beas2B cell line (Figure 3B, right panel), where both the full-length and 305-bp products were amplified. Direct nucleotide sequencing confirmed the shorter amplicon to be CD1D with a spliced α1 exon, which we called “V1” (Figure 3B, bottom panel). To ensure that V1 is not a non-specific amplification, RT-PCR using the C/C primer was repeated on cDNA reverse transcribed using a CD1D-specific primer (GGACGCCCTGATAGGAAGTT) rather than oligo dT. The latter also showed the presence of V1 (Figure 3B, right panel).

For “D/D” and “D/E” primers, the forward primer in both sets originates in the first half of the α2 exon, while the reverse primers *Rev.* D and *Rev.* E originate in T (tyrosine tail) and 3′-UTR of exon 7, respectively (Figure 3A). RT-PCR with “D/D” amplified six products with varying lengths (Figure 3C). Only one (Non-Spliced; NS) corresponded to expected non-spliced transcript (636-bp). To examine whether the other amplified DNAs were spliced forms of CD1D, the bands were cut out from the gel and nucleotide sequence determined. This confirmed all as PCR products for CD1D (“V2–6”). In addition, sequencing revealed the splice junctions for three of the variants: “V4”, “V5”, and “V6” (Figure 3D). V4 and V5 were deficient in α3 (279 bases) and α3-TM (379 bases), respectively; whereas “V6” represented a variant with double splicing events, trimming out the second half of α2 (last 133 out of 279 bases) and most (274 out of 279 bases) of the α3 exon.

**Figure 4:**
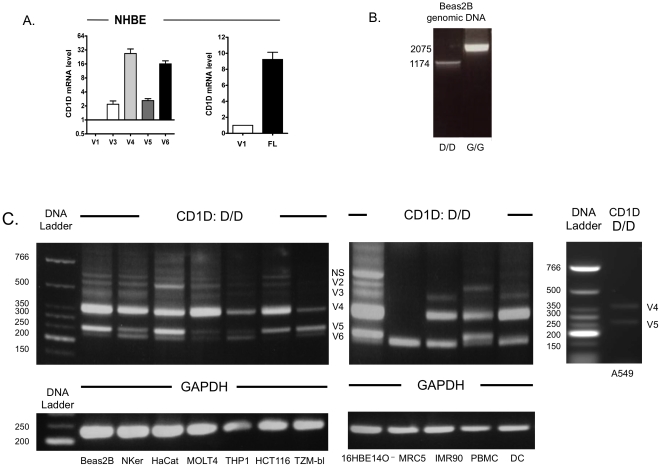
Relative expression of CD1D transcripts in human respiratory epithelial cells and cell-type pattern of variant expression. Panel A: Relative abundance of different CD1D variants by qPCR in NHBE cells compared to “V1”(left), and between “V1” and full length (FL). Panel B: PCR using “C/C” and “D/D” primers in genomic DNA from Beas2B cell line. Panel C: Pattern of expression for CD1D variant in different human cell lines and primary cells; GAPDH was used as a control.

To compare the relative amount of variants, qPCR using SYBR Green methods or Taqman probe was carried out for “V1”, “V3–6”, using the primer and probe sets described in Table 1. Multiple primers for “V2” produced late Ct on amplification plot and were inconsistent. We believe this reflected very low level of gene expression and therefore did not test V2's relative levels. We tested these primers in NHBE primary cells (Figure 4A, left panel). This allowed us to confirm the variants in primary human bronchial epithelial cells and also show that relative to the alpha-1 deficient splice variant (“V1”) (Figure 4A), “V4” and “V6” were most highly expressed. Relative to the full length transcript (FL), “V1” was 9.2 fold lower (Figure 4A, right panel). Characteristics and potential implications of “V1–6” are summarized in Table 2.

**Table 2:** Predicted properties of CD1d proteins coded by different mRNA transcripts in human respiratory epithelial cells.

Variant	Spliced sequence	Frame shift	Stop codon position	Membrane insertion	Ag presentation	Predicted MW (kDa)
V1	α1	In-frame	Unchanged	Likely	Unlikely	27.2
V2	TM	Out-of-frame	78^th^ base in exon 7	Unlikely	Likely	36.1
V3	Part of α2	Out-of-frame	1^st^ base in α3 exon	Unlikely	Unlikely	21.4
V4	α3	In-frame	Unchanged	Likely	Likely	27.4
V5	α3-TM	Out-of-frame	78^th^ base in exon 7	Unlikely	Likely	25.8
V6	Part of α2+α3	Out-of-frame	25^th^ base in TM exon	Unlikely	Unlikely	18.9
NS	−	−	−	+	+	37.7

V1–6: CD1D spliced variants; NS: Non-spliced.

To exclude possibility of DNA defects as the source for these variants, genomic DNA from Beas2B were amplified using two of the primers and showed to have only one band (Figure 4B).

### CD1D spliced variants are expressed on epithelial cell lines but not in antigen presenting cells, and fibroblast lines

Although we have shown existence of CD1D variants in human bronchial epithelial cells, it is unclear if the same set of variants is observed in other cells. Unlike other CD1 family members (CD1A, CD1C, and CD1E), CD1D splicing has only been marginally studied in two reports [20], [21]. One study found eight variants in peripheral blood mononuclear cells (PBMCs), but using nested PCR which reduced the reliability of the results [20]. Neither study compared the expression of the spliced variants across different cells. To address this, we examined CD1D variants in several cell lines representing epithelium from various sites (Beas2B, 16HBE14O−, NKer, HaCat, HCT116 and TZM-bl; sources described in Methods) compared to antigen presenting cells (THP-1), T cells (MOLT4) and fibroblasts (MRC5 and IMR90). Using a primer set that identified variants “V2–6” (“D/D”), we found similar splicing patterns among bronchial epithelial cells 16HBE14O^−^ and Beas2B, normal keratinocytes (NKer) and colon epithelial cells (HCT116) (all expressed full length and “V2” to “V6” variants). This suggests potential similarity in splicing machinery at resting state across different mucosa-lining epithelial cells, though cancerous transformation or immortalised nature of cell lines may account this phenomenon The fibroblasts only expressed “V6” (MRC5) and “V3”, “V4” and “V6” (IMR90). The primary cells PBMC and dendritic cells (DCs) also expressed all variants but possibly to a different relative amounts (Figure 4D). The α3–deficient CD1D variant (“V4”), when present, was the most intensely PCR-amplified transcript in all cell types.

## Discussion

In this paper, we show that CD1d is expressed on bronchial epithelial cells, both in primary and airway epithelial cell lines, and that there are at least six spliced variants likely specific to epithelial cells. We felt that examining the function of these epithelial CD1d transcripts was beyond the scope of this paper so it is unclear currently how these proteins function in the context of the lungs. However, it is possible to speculate on the potential implications of CD1d expression on these structural cells. Due to the large surface area of the airway epithelium, one prospect of CD1d expression here is the rapid presentation of both self and exogenous glycolipids to infiltrating NKT cells. This could maintain NKT cells proliferation in situ and prolong their protective effect in the lungs. The opposite is also possible – that of enhancing NKT cell's harmful effect e.g. airway hyper-responsiveness observed in murine models of asthma. In the ovalbumin asthma model, CD1d^−/−^ mice do not develop airway hyper-responsiveness [9], [22], [23], [24].

A potentially interesting part of our results is the observation of the same profile of transcripts in epithelial but not haematopoietic cells. The epithelial cell lines from skin, lungs, cervix and colon showed “V2” to “V6” expression (“V2” and “V3” very faint in the cervical epithelial cell line, TZM-bl). The exception to this is A549, a type II alveolar epithelial cell line which only expressed “V4” and “V5”. Type II alveolar epithelial cells have the unique property of producing surfactants, which regulate the surface tension of the alveoli and also to contribute to host defence. In the latter, two subsets of surfactant (SP-A and SP-D, part of collectins family) act as opsonins to facilitate elimination of pathogens by alveolar macrophages [25]. The CD1D transcripts on these cells (“V4” and “V5”, discussed later as potential ß2m-independent and soluble forms respectively) could serve as innate host defence molecules rather than antigen-presenting molecules.

Against expectation, we observed only very low/undetectable levels on full length mRNA in PBMC and DCs (Figure 4C). There have been very few studies on the variety of CD1D mRNAs in these cells and their relative expression to the full-length transcript. Kojo et al mentioned existence of multiple CD1D transcripts in PBMCs, however, the level of expression was so low that they required nested PCR to identify this; only three healthy volunteers were studied, among these, one had extremely low/undetectable levels of non-spliced full-length CD1D. Our low expression is in keeping with their finding, and the low/undetectable expression by one-round of PCR in MOLT-4 and THP cells support the finding in primary hematopoietic cells Since the CD1d protein has been identified in these cell populations, one possibility is that the mAbs used detected proteins translated by the variants rather than the full length CD1D.

Some potential functions of the different variants could be gleaned from knowledge of the various regions that are spliced (Table 2). Lipid-binding groove in CD1d is formed by α1 and α2 domains [26], [27]. The protein for “V1”, deficient in α1, is unlikely to participate in antigen loading and presentation. Hydrophobic amino acid residues encoded by transmembrane (TM) exon are required for insertion of the CD1d protein into plasma membrane. The TM exon is spliced out in “V2” and “V5”. In addition, “V3” and “V6” as a consequence of shift in reading frames contain early stop codons in α3 and TM, respectively. So, it is likely that proteins coded by these variants will be either secreted (soluble isoform) or retained intracellularly. There is precedence for this - Woolfson *et al* showed expression of CD1A and CD1C TM-deficient transcripts, and detected corresponding soluble protein isoforms [28]. Similarly, soluble HLA-G, an MHC class I molecule which has limited polymorphism and shares high structural similarity with CD1D [29], is encoded by a TM-deficient variant [30], [31], and was recently shown to activate NK cells via NFκB signalling [32].

“V4” is the most highly expressed transcript in NHBE cells; and is potentially an interesting variant which could have epithelial-specific function. Its protein product is predicted to lack ß_2_m binding ability but it has intact TM and cytoplasmic tail (T) exons, so it can produce a cell surface-expressed β_2_m-free CD1d isoform. On human intestinal epithelial cells, the major form of CD1d is a non-glycosylated, ß_2_m-independent molecule, although these cells also express a ß_2_m-associated, fully glycosylated form of CD1d [30]. The importance of ß_2_m association with CD1d with respect to the CD1d biosynthetic pathway is not established. Association of ß_2_m with CD1d could have a role in regulating the extent of CD1d glycosylation and the maturity of the attached carbohydrate side chains [33]. Although, in most cases, forms of MHCI protein that do not associate with ß_2_m do not fold their antigen presenting domains properly, cells from ß_2_m -deficient mice are able to stimulate proliferation of CD1d-restricted T cell clones [34], suggesting presence of a CD1d variant that do not rely on ß_2_m for antigen presentation and function.

Regardless of the transcripts, identification by antibodies confirms the presence of protein on the surface of bronchial epithelial cells. The CD1d antibody, clone 42, has been widely used as a blocking antibody to prevent activation of NKT by CD1d-expressing cells [35], [36], [37], therefore flow cytometry results verify presence of (at least) one functional CD1d (likely full length protein) with the ability to associate with ß_2_m on the surface human bronchial epithelial cells.

In summary, we have identified for the first time, CD1D expression on human bronchial epithelial cells and detection of at least some of the variant protein by CD1d monoclonal antibodies. This provides a basis for further investigations for the role of this molecule in lung immune responses.

## Materials and Methods

### Cells and culture conditions

Fresh primary human bronchial epithelial cells were obtained from the primary bronchus of a patient undergoing bronchoscopy for a localised lung tumour. Samples were obtained on the contra-lateral (normal) side to the tumour, by brushing the primary bronchus using a bronchoscope brush (KeyMed fiberoptic bronchoscope; Olympus; Japan). The cells were dislodged into complete RPMI-1640 (Invitrogen, UK) by rigorous agitation of the brush within the container and used within 6 hours. The patient provided written informed consent and the study was approved by the Oxfordshire Research Ethics Committee.

Primary normal human bronchial epithelial cells (NHBE cells) were purchased from Lonza (Walkersville, Inc), and grown in serum-free and hormone and growth factor supplemented bronchial epithelial growth media (BEGM) (Lonza, Walkersville, Inc.), under submerged conditions.

All cell lines were purchased from the American Type Culture Collection (Manassas, VA), apart from NKer, a human keratinocyte line immortalized using human papilloma virus-16 E6 protein which was a kind gift from Dr E O'Toole, (Northwestern University Medical School, Chicago, USA). Beas-2B was cultured in F12 Nutrient Mixture Kaighn's Modification (F12K); A549 (human type II alveolar epithelial cell line), 16HBE14O^−^ (differentiated SV40-transformed human bronchial epithelial), TZM-bl (CD4-expressing adenocarcinoma-derived human cervical epithelial cell line), HaCat (spontaneously immortalised human keratinocyte), HCT116 (human colon carcinoma epithelial) and NKer were maintained in Dulbecco's modified Eagle medium (DMEM) (Invitrogen, Carlsbad, CA). Jurkat (an immortalized T cell line), THP1 (a human monocytic/macrophage cell line) and MOLT4 (a human leukemic T lymphoblast cell line) were cultured in RPMI-1640. All cell culture media were supplemented with 10% FBS (Invitrogen), 2 mM L-glutamine (Invitrogen), 50 U/ml penicillin G, and 50 mg/ml streptomycin (Invitrogen) (complete RPMI 1640). Cells were cultured at 37°C and 5% CO_2_.

Monocyte-derived dendritic cells (DCs) were generated as previously described [35]. All DCs were checked for maturation markers (CD83, CD86, and HLA-DR; eBioscience, USA) and determined to be CD1b^+^ and CD1d^+^ and CD14^−^ at time of use.

### DNA preparation, RNA isolation and cDNA preparation

Genomic DNA was purified using DNeasy Blood & Tissue Kit (Qiagen, UK). Total RNA was extracted by lysing the cells in 1 ml TRIzol reagent (Ambion, UK) or using RNeasy Mini Kit (Qiagen, UK), following the manufacturers' protocols. After extraction with either protocol, RNA was incubated with DNase I (Qiagen, UK) for 15 minutes at room temperature to remove residual contaminating DNA, and the enzyme inactivated by heating at 65°C for 5 minutes. Isolated total RNA was reverse transcribed using SuperScript® Reverse Transcriptase III kit (Invitrogen, UK), according to manufacturer's protocols. cDNA synthesis was primed by mixing 1 µl of either oligo dT (50 µM), or gene-specific primer (1 µM), and 1 µl of dNTP mix (10 mM) with up to 5 µg total RNA.

### Reverse transcription PCR and real time quantitative PCR

Primers and probes were designed using either Primer Express® software version 3.0 (Applied Biosystems, UK) or the online Primer3 application (http://frodo.wi.mit.edu/primer3/). For real time PCR analysis, the melting temperatures of probes were 10°C higher than the corresponding primers, and the amplicons had a length of 50–150-bp.

For RT-PCR, two microlitres cDNA was added to 48 µl reaction mix containing 2 U FastStart Taq DNA Polymerase (Roche, UK), 5 µl 10× PCR buffer, 2 mM MgCl_2_, 1 µM of each dNTP and 1 µM of forward and reverse primers and amplified by GeneAmp® PCR System 2700 thermal cycler (Applied Biosystems, UK).

Real time PCR was performed using 7500 Fast real time PCR system (Applied Biosystems, UK). Reactions contained 2 µl cDNA added to 10 µl 2×Universal or SYBR Green Master Mix (Applied Biosystems, UK) and appropriate CD1d forward and reverse primers, and probes where used (Eurogentec, UK). PCR conditions for SYBR Green assay were as followed: 50°C for 2 minutes, 95°C for 10 minutes and 45 cycles of 95°C for 15 seconds and 60°C for 1 minute. Taqman assay conditions were optimised at 95°C for 20 seconds and 45 cycles of 95°C for 3 seconds and 60°C for 30 seconds.

The results were quantified using 2^−ΔΔCt^ method [38], where ΔCt (Ct_CD1D variant_−Ct_housekeeping gene_) was calculated initially, and then ΔΔCt was obtained by subtracting ΔCt of reference CD1D variant (e.g. V1) from that of a given CD1D variant (e.g. V3, V4, etc). Comparable amplification efficiencies for both housekeeping genes and CD1D variants was verified by serial dilution of two highly abundant CD1D variants against HPRT housekeeping genes.

Primer and probe sequences are depicted in Table 1.

### Immunofluorescence microscopy

Cells were cytospun and fixed in 4°C cold acetone and non-specific antibody-binding sites were blocked by incubation with blocking buffer (5% FCS, 1% BSA in PBS); and stained with pancytokeratin (PCK) mAb (Abcam, UK), or β-tubulin IV (BioGenex, UK), and then Alexa Fluor® 633-conjugated goat anti-mouse IgG (Invitrogen, UK).

### DNA sequencing

Purified PCR products were sequenced using the BigDyeR Terminator v3.1 cycle sequencing kit (Applied Biosystems, UK) and analyzed using a 3730 ABI capillary electrophoresis system (Applied Biosystems, UK).
